# Subcutaneous abscess due to empyema necessitans caused by *Porphyromonas gingivalis* in a patient with periodontitis

**DOI:** 10.1016/j.idcr.2022.e01458

**Published:** 2022-02-21

**Authors:** Akane Tanaka, Mariko Kogami, Yoko Nagatomo, Yukihisa Takeda, Hiroya Kanzawa, Yohei Kawaguchi, Shotaro Ono, Kinya Furukawa, Hiroyuki Nakamura, Kazutetsu Aoshiba

**Affiliations:** a Department of Respiratory Medicine, Tokyo Medical University Ibaraki Medical Center, 3-20-1 Chuou, Ami, Inashiki, Ibaraki 300-0395, Japan; b Department of Respiratory Medicine, Tokyo Medical University, 6-7-1 Nishishinjuku, Shinjuku-ku, Tokyo 160-0023, Japan; c Department of Thoracic Surgery, Tokyo Medical University Ibaraki Medical Center, 3-20-1 Chuou, Ami, Inashiki, Ibaraki 300-0395, Japan

**Keywords:** Empyema necessitans, Porphyromonas gingivalis, Subcutaneous abscess, Lung abscess, Periodontitis

## Abstract

Empyema necessitans is a rare empyema complication characterized by an extension of empyema out of the pleural space into the subcutaneous tissues of the chest wall. We herein report a case of empyema necessitans that presented as a subcutaneous chest wall abscess caused by *Porphyromonas gingivalis* (*P. gingivalis*), an important anaerobic periodontal pathogen, in a 74-year-old woman with periodontitis. The patient was admitted to our hospital with a painful soft tissue mass in the chest wall extending from a subpleural lung abscess associated with empyema. Exploratory percutaneous puncture and aspiration of the chest wall mass yielded foul-smelling chocolate-colored pus, which was found to be caused due to infection with *P. gingivalis*. Treatment with antibacterials resulted in a relapse of empyema necessitans requiring a second admission 1 month later. An additive treatment with surgical open drainage and decortication of the subcutaneous abscess successfully cured the abscess. Physicians must be aware of emphysema necessitans as an etiology of a chest wall mass and should consider periodontitis as a source of infection.

## Introduction

*Porphyromonas gingivalis* (*P. gingivalis*) is a well-recognized pathogen causing periodontitis. It was reportedly detected in 86% of subgingival plaque in patients with periodontitis [1]. Although this oral commensal rarely causes extraoral infection, it produces a wide array of virulence factors, including proteolytic enzymes, capsule, lipopolysaccharide, and fimbriae, which can cause tissue destruction, severe inflammation, and sometimes abscess formation [2]. Empyema necessitans is a rare complication of thoracic infection, characterized by an extension of empyema out of the pleural space into the subcutaneous tissues of the chest wall [3], [4]. To the best of our knowledge, we report the first case of a patient with periodontitis who developed a chest wall abscess due to empyema necessitans caused by *P. gingivalis*.

## Case report

**Fig. 1:**
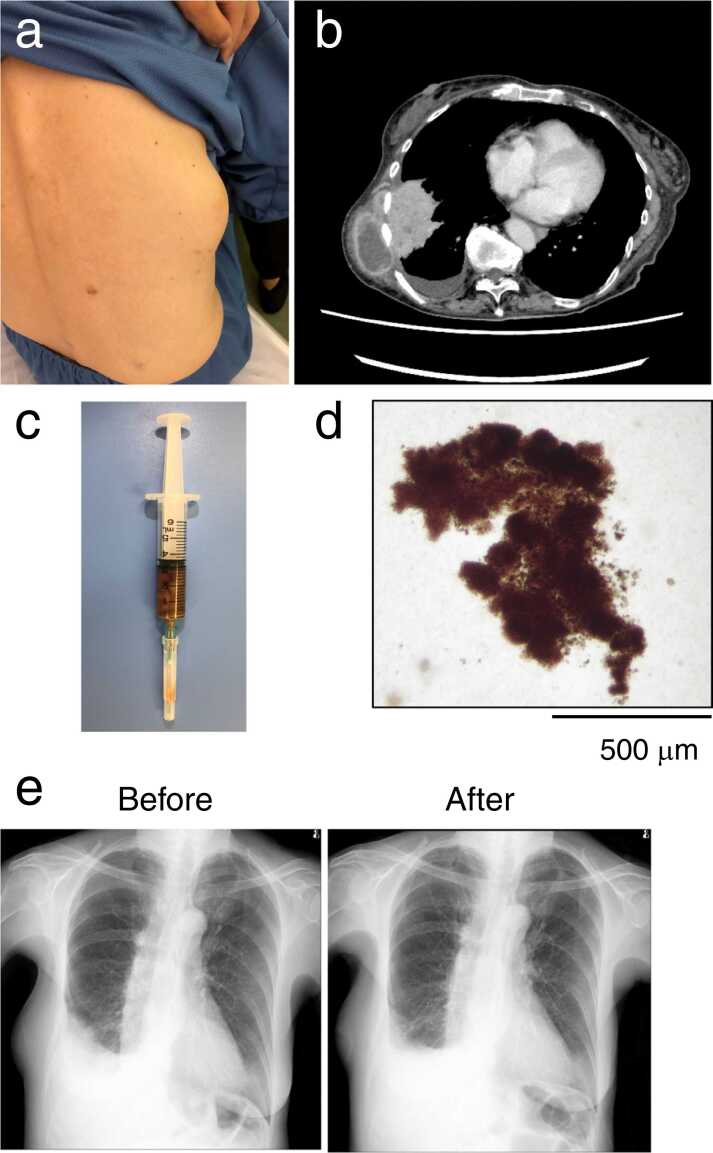
Clinical findings during the first admission of the patient. (**a)** Bulging mass on the right region of the chest wall. (**b**) Initial contrast-enhanced computed tomography (CT) scan of the chest revealed a subpleural lung abscess and pleural effusion with extension to the subcutaneous mass. (**c**) Chocolate-colored pus obtained by an exploratory percutaneous puncture and aspiration of the subcutaneous abscess. (**d**) Microscopic examination of the pus revealed brown-black-pigmented bacterial granules. (**e**) Chest X-ray taken before and after treatment with antibiotics showed improvements of chest wall mass and lung opacification.

A 74-year-old woman without any history of smoking or drinking was admitted to our hospital due to a painful chest wall mass that was first noticed 1 month earlier and increased in size. She underwent gastrectomy for gastric cancer 15 years previously and was undergoing treatment for periodontitis for 3 months. Physical examination revealed that she had fever (38.4 °C) and a 10-cm soft, elastic, and tender bulge in her right lateral chest wall (Fig. 1a). She had dry cough, but wheezes or lung crackles and abnormal heart sounds were not observed upon auscultation. Blood test results revealed leukocytosis (12,300 cells/μL with 87% neutrophils) and a high C-reactive protein (CRP) level (27.5 mg/dL). A chest computed tomography (CT) scan showed a subcutaneous abscess with a diameter of 9 cm extending from a right lower subpleural lung abscess with a diameter of 8 cm accompanied by empyema (Fig. 1b).

An exploratory percutaneous puncture and aspiration of the subcutaneous abscess yielded a foul smelling chocolate-colored pus (Fig. 1c). Microscopic examination revealed that the pus contained brown-black-pigmented bacteria granules (Fig. 1d). Based on these findings, the diagnosis of subcutaneous abscess extending from the lung abscess and associated empyema, referred to as empyema necessitans, was made, and treatment with intravenous ampicillin/sulbactam (12 g/day) was initiated. Gram-stained smears of the pus revealed gram-negative rods, and a pure culture of *P. gingivalis* was grown on anaerobic culture. Aerobic culture yielded no growth. Two teeth were extracted due to severe periodontitis, which was suspected to be a source of pulmonary infection by *P. gingivalis*. After the initiation of antibacterials, the patient’s condition improved with a considerable resolution of abnormal shadows on chest X-ray (Fig. 1e), and also serum CRP levels were normalized. Treatment with antibacterials was discontinued after 28 days and the patient was discharged 30 days after admission.

**Fig. 2:**
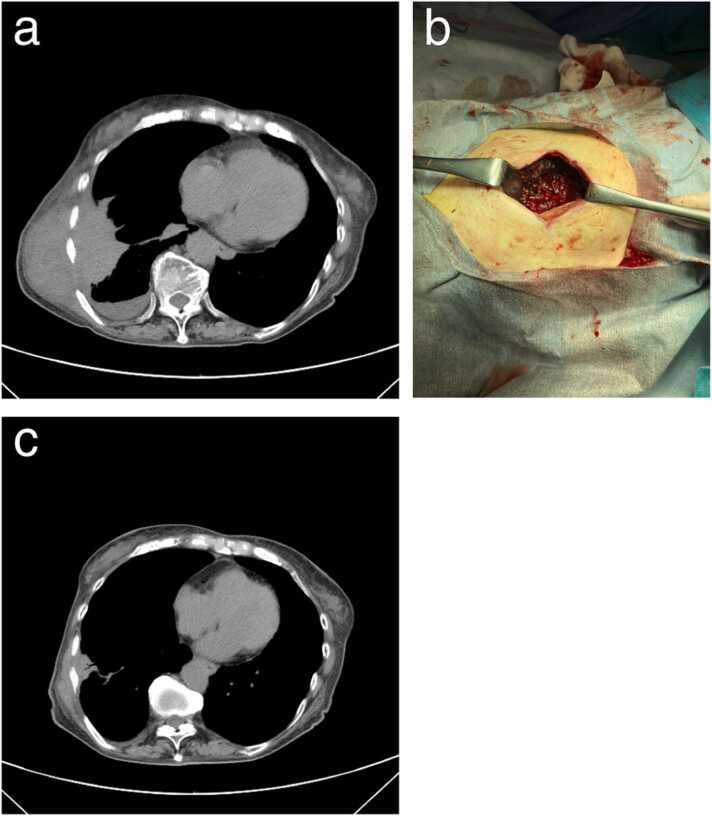
Clinical findings during the second admission of the patient. (**a**) Plain CT scan of the chest revealed a relapse of empyema necessitans, characterized by increases in subpleural lung abscess, pleural effusion, and subcutaneous abscess. (**b**) A photograph of the surgical incision into the subcutaneous mass taken on day 4 of the second admission. (**c**) A plain chest CT scan performed after treatment with antibiotics and open drainage with debridement showed marked improvement of lung abscess, pleural effusion, and subcutaneous abscess.

Twenty-nine days after discharge the patient was readmitted with a complaint of regrowth of the chest wall mass with tenderness. Blood test findings revealed elevated CRP level (10.5 mg/dL). A chest CT scan showed increased size in the lung and subcutaneous abscesses with an increased collection of pleural fluid (Fig. 2a**)**. A diagnosis of empyema necessitans relapse was made, and treatment with intravenous ampicillin/sulbactam (12 g/day) was restarted. The puncture-aspirated pus from the subcutaneous abscess again yielded a pure culture of *P. gingivalis*. The broad-range bacterial 16 S rRNA PCR assay for the pus sample also identified *P. gingivalis* as a single pathogen. On day 4 of the second admission, a surgical incision of the subcutaneous abscess with debridement was performed (Fig. 2b), resulting in the drainage of a large-volume purulent material. This led to marked clinical and radiological resolution (Fig. 2c). On day 16 of the second admission, treatment with intravenous ampicillin/sulbactam was switched to an oral dose of amoxicillin/clavulanate (1 g/day), which was continued after discharge on day 23. Follow-up at 2 months after discharge showed no recurrence with continuation of the antibacterials.

## Discussion

*P. gingivalis* is a gram-negative oral anaerobe forming a black-pigmented colony and is considered to be a major pathogenic agent causing periodontitis. Furthermore, *P. gingivalis* can be aspirated into the lungs, colonize respiratory airways, and sometimes cause aspiration pneumonia [5]. Besides direct infection of the lungs, other mechanisms of pneumonia pathogenicity of *P. gingivalis* have been proposed [6]. For example, gingipain, a trypsin-like protease secreted by *P. gingivalis* [1], has been shown to upregulate epithelial cell expression of platelet-activating factor receptor, which mediates infection of airway epithelium by pneumonia-causing bacteria, such as *Streptococcus pneumoniae* and *Haemophilus influenzae* [7]. Furthermore, *P. gingivalis*, even when dead, has been reported to induce the secretion of proinflammatory cytokines, including IL-6 and IL-8, from human respiratory epithelial cells, thereby potentially promoting the development of pneumonia [8].

Although extraoral abscess formation by *P. gingivalis* is rare, multiple cases of brain abscesses have been reported [9], [10], [11]. Other extraoral abscesses include appendicitis, otitis media, thoracic empyema, and lung abscess [12]. Subcutaneous abscess due to *P. gingivalis* infection is very rare. Only one case study was found in the literature that describes facial subcutaneous abscess caused by *P. gingivalis*, which developed as a primary infection site without obvious primary site, in a patient undergoing chemotherapy for lung cancer [13]. To the best of our knowledge, this case report is the first to document chest wall subcutaneous abscess caused by *P. gingivalis*, as a manifestation of empyema neccessitans, in which the lung parenchyma, pleura, and chest wall were all involved.

Bacterial pneumonia or lung abscess can cause a parapneumonic empyema. Empyema neccessitans is caused by the extension of empyema into the chest wall, which occurs along the path of least resistance in the parietal pleura as a result of increased pressure within pleural loculation, chronic inflammation, and necrosis with erosion [3], [4]. The most commonly reported pathogens are *Mycobacterium tuberculosis* and *Actinomyces* species [3], [4], [14]. Other rare causal organisms include *Staphylococcus*, *Streptococcus*, and *Nocardia* [3], [4]. Bacterial virulence is likely to contribute to the formation of subcutaneous pleural fistula because organisms must dissect the parietal pleura into the chest wall to develop empyema neccessitans. In this context, *P. gingivalis* notably produces gingipains, which largely contribute to extracellular matrix destruction and dysregulated inflammation [2], [15].

Microscopic observation of the pus sample obtained from subcutaneous abscess has revealed the presence of black-colored granules (Fig. 1d), which were different in color from yellow-colored, sulfur granules reported in actinomycosis infection. The granules are likely to be a collection of bacteria surrounded by inflammatory debris, as *P. gingivalis* is known to produce black-pigmented colonies on an anaerobic blood agar plate [1], [2]. Abscesses are often polymicrobial infections caused by aerobes and anaerobes. However, aspirates from the subcutaneous abscess in our patient yielded pure culture of *P. gingivalis* without any other bacteria identifiable on the broad-range bacterial 16 S rRNA PCR assay.

In this case, treatment with antibiotics alone resulted in a relapse of empyema necessitans requiring a second admission 1 month later. An additive treatment with surgical open drainage and decortication of the subcutaneous abscess was found to be useful. Due to the small volume of pleural effusion, thoracostomy drainage of pleural empyema was not performed.

In conclusion, this case suggests that *P. gingivalis* can cause lung abscess, empyema, and emphysema necessitans. Periodontitis should be considered while examining patients with emphysema necessitans presenting as a chest wall mass. A combination of antibiotics and open drainage with decortication was found to be useful for treatment.

## Consent

Written informed consent was obtained from the patient for the publication of this case report and accompanying images. A copy of the written consent is available for review by the Editor-in-Chief of this journal on request.

## CRediT authorship contribution statement

**Akane Tanaka:** Writing – original draft preparation; **Mariko Kogami:** Writing – review and editing; **Yoko Nagatomo:** Writing – review and editing; **Yukihisa Takeda:** Writing – review and editing; **Hiroya Kanzawa:** Writing – review and editing, **Yohei Kawaguchi:** Writing – review and editing; **Shotaro Ono:** Writing – review and editing; **Kinya Furukawa:** Writing – review and editing; **Hiroyuki Nakamura:** Writing – review and editing; **Kazutetsu Aoshiba:** Writing – original draft preparation and Writing – review and editing, Supervision.

## Author statement

All authors have made significant contributions to the planning, conduct, and reporting of the work described in this article. All authors have read and approved the submission of this final manuscript.

## Funding

This research did not receive any specific grant from funding agencies in the public, commercial, or not-for-profit sectors.

## Ethical approval

This case report meets the standards of the Tokyo Medical University Ethical Committee. All personal identifiers were removed from the manuscript.

## Data Availability

The data that support the findings of this study are available on request from the corresponding author. The data are not publicly available due to privacy or ethical restrictions.
